# Masticatory performance and masticatory behavior in individuals with eating disorders: a pilot study

**DOI:** 10.3389/froh.2024.1456418

**Published:** 2024-11-21

**Authors:** Abhishek Kumar, Linda Munirji, Billy Langlet, Nagihan Bostanci, Anastasios Grigoriadis

**Affiliations:** ^1^ Department of Dental Medicine, Karolinska Institutet, Huddinge, Sweden; ^2^ Academic Center for Geriatric Dentistry, Stockholm, Sweden; ^3^ Department of Medicine, Karolinska Institutet, Huddinge, Sweden

**Keywords:** masticatory function, jaw tracker, chewing, anorexia nervosa, masseter muscle, viscoelastic food

## Abstract

**Aim:**

The brief report aims to evaluate masticatory performance and components of chewing behavior in people with anorexia nervosa and compare it with a reference group of individuals with no history of eating disorders.

**Material and methods:**

Eighteen women participated in the study: nine with anorexia nervosa (age 20.2 ± 5.9) and nine as controls in a reference group without anorexia nervosa (age 23.6 ± 0.9). Masticatory performance was assessed with a food comminution test. The participants also ate (chewed and swallowed) a second test food while their jaw movements were recorded to evaluate their chewing behavior. The number of comminuted pieces during the food comminution test, chewing cycles, chewing duration, and components like occlusal, and jaw opening/closing duration, were evaluated.

**Results:**

In comparison to the reference group the anorexia nervosa patients performed poorly in the food comminution test (*P* = 0.007), and also chewed the test food significantly longer (*P* = 0.004) and with significantly more chewing cycles (*P* = 0.014). The results also showed a significantly longer jaw-closing duration in the anorexia nervosa group compared to the reference group (*P* = 0.021). However, there were no significant differences in either the jaw opening or the occlusal duration between the two groups.

**Conclusion:**

Overall, the results of the study indicate that the anorexia nervosa group shows signs of poor masticatory performance and altered chewing behavior compared to the reference group of individuals with no history of eating disorders.

## Background

Chewing function is a significant “contributor” to swallowing and digestive function and may affect the nutritional status of humans (1). A chewing sequence typically consists of a series of chewing cycles that begin when food is placed in the mouth and ends when the bolus is swallowed. Chewing function is affected by several factors including age, number of posterior and antagonistic tooth contacts, saliva flow rate, type of prosthesis, occlusion, etc. (2). It is reported that a high eating rate, large bite size, and short food oral processing time have all been associated with an increased risk of overeating and weight gain (3). Conversely, chewing food thoroughly can release nutrients from food more efficiently, subsequently affecting gut signaling, physical actions, and ultimately digestive and absorptive processes (4). Furthermore, it is suggested that changes in eating behavior also cause changes in moods associated with eating disorders (5).

A person's oral health and oral (chewing) function are important indicators of his/her general health and quality of life (1, 6). The process of chewing food is regulated by the central nervous system and influenced by signals from the peripheral receptors in the oral cavity (7). The central nervous system is responsible for generating the basic rhythm of chewing, while orofacial receptors provide continuous feedback to the brain about the changing properties of the food. Chewing food causes changes in mood by engaging the serotonin neurons of the dorsal raphe nucleus and their projections to the orbitofrontal and prefrontal cortex (8, 9). This is a subset of the extended neural circuitry that is active when the food is consumed (10).

Healthy eating habits can be affected by impaired chewing efficiency due to either poor dentition or impaired sensorimotor regulation (11). If food is not chewed long enough, the particles are too big and fail to cohere. Whereas, if food is chewed for too long, the bolus becomes too wet and falls apart making it difficult to swallow (12). Further, it is suggested that the neural network associated with chewing may also regulate swallowing initiation. Hence, it is hypothesized that maintaining an ideal “chewing-swallowing rhythm” could be important for healthy eating and general well-being.

Examining chewing function and chewing behavior in patients with Anorexia Nervosa and other eating disorders can be important due to the significant impact these conditions have on overall health and well-being (13). Anorexia nervosa is characterized by severe caloric restriction, intense fear of weight gain, and distorted body image, leading to malnutrition and various health complications (14). These patients often suffer from compromised oral health, including xerostomia, dental hypersensitivity, increased dental caries, and periodontal disease, which can impair masticatory performance (15–18). Additionally, the psychological state of individuals with anorexia nervosa influences their masticatory behavior, leading to prolonged chewing or avoidance of certain food textures due to fear of weight gain or discomfort (19). Stress further complicates this, as it can alter chewing frequency and food intake, suggesting that chewing might play a role in the regulation of eating behaviors (13). Understanding these behaviors is essential for developing targeted interventions to improve both oral health and nutritional outcomes.

Normalizing eating behavior has been proposed as an effective intervention for the management of eating disorders, including anorexia nervosa (20). This approach focuses on establishing regular (normalization), healthy eating patterns and regulating food intake during meals (20, 21). However, it is suggested that more comprehensive methods are needed to study the “microstructure” of food ingestive behavior (5). Therefore, in the current study, we aimed to evaluate the masticatory performance and analyze the “micro-characteristics” of chewing behaviors in people diagnosed with anorexia nervosa (eating disorder) and compare them with individuals with no history of eating disorders. Specifically, we hypothesize that individuals with anorexia nervosa may have poor masticatory performance and altered masticatory behavior in terms of jaw movements, and other micro-characteristics of masticatory behaviors compared to individuals with no history of eating disorders.

## Methodology

The study was approved by the Regional Ethics Review Board in Stockholm, Sweden (Dnr 2020-03646), and was performed in compliance with the Declaration of Helsinki, and Good Clinical Practice. The study was conducted at the Department of Dental Medicine, Karolinska Institutet in collaboration with the Mandometer Clinics, Huddinge, Sweden.

### Study participants

Eighteen women participated in the pilot study comprising an experimental group (*n* = 9, age = 20.2 ± 5.9) and a reference group (*n* = 9, age = 23.6 ± 0.9). To be enrolled in the study, both the patients admitted to the Mandometer Clinics, and the participants in the reference group were required to attend an information meeting during which researchers provided information about the study. Participants from both groups were recruited if they provided written informed consent to participate in the study. The participants in the experimental group were patients diagnosed with anorexia nervosa and admitted to “in-patient” or “outpatient care,” depending on the severity of the disorder. The participants in the reference group were healthy women with no history of eating disorders, who volunteered to participate in the study. The participants in the reference group were recruited by posting flyers and advertisements in and around the university campus. The inclusion criteria for the reference group were no current or previous history of eating disorders. The participants in both groups were excluded if they reported any signs or symptoms of orofacial pain or temporomandibular disorders, current/previous prosthodontic or endodontic treatment, gross malocclusion of the anterior or posterior teeth, including gross overjet, overbite, crossbite or any other signs or symptoms related to impaired masticatory function. While the BMI for the anorexia nervosa was obtained from their medical records the BMI for the reference groups was calculated based on the self-reported height and weight by the participants.

### Experimental protocol

The participants from both groups participated in a single experimental session of 30–60 min. The experimental session was conducted in a quiet and well-lit room. The experimental session was composed of two clinical tests: (i) masticatory performance and (ii) masticatory behaviors. During both tests, the participants were asked to chew two pieces of hard viscoelastic test food, one to evaluate their masticatory performance, and the other to evaluate the associated masticatory behavior as described below.

### Masticatory performance

The masticatory performance was assessed by a hard viscoelastic food comminution test (22–24). Accordingly, a cylindrical, hard viscoelastic test food (dimensions: Diameter 20 mm × Height 10 mm; weight: 4.2 ± 0.1 grams) was prepared following a standard recipe as described earlier in detail (24, 25). During the test, the participants were asked to place the test food in their mouth between the tongue and the palate. Thereafter the participants were instructed to eat the test food while the examiner observed the participants and silently counted the number of chewing strokes. After participants had chewed on the test food for ten chewing strokes they were abruptly interrupted and instructed to stop chewing and expectorate the test food into a white petri dish. The expectorate of the chewed test food was photographed and the number of chewed pieces of the test food was analyzed with image analysis software (ImageJ) following previous studies (22). The number of comminuted particles was considered as the marker of masticatory performance. The higher the number of comminuted particles, the better the chewing performance.

### Masticatory behavior

The masticatory behavior was assessed by the analysis of jaw movements and jaw muscle activity during the behavioral tasks following the previous studies (26, 27). Accordingly, jaw movements in three-dimension along with the electromyographic (EMG) activity of the right and left masseter during the behavioral tasks were recorded with a custom-built device (Umeå University, Physiology Section, IMB, Umeå, Sweden) (25). A small magnet measuring 10 × 5 × 5 millimeters was attached to the labial surface of the lower central incisors using dental composite and cured with an LED lamp. The participants were then comfortably seated in an office chair with a light frame that rested on the bridge of their nose and was secured around the head like a pair of glasses. The frame attached to the head was equipped with an array of magnetic sensors that could monitor the movement of a magnet attached to the teeth in three dimensions with a high accuracy of about 0.1 mm and a bandwidth of DC—100 Hz. The setup was comfortable and allowed the participants to maintain their typical head movements while performing the behavioral tasks.

The experimental setup and the subsequent analysis of masticatory behavior have been previously reported (28–30). EMG activity from the masseter muscles was recorded by placing a pair of electrode-shielded preamplifiers (bandwidth 6 Hz to 2.5 kHz) mounted on the skin directly above the surface electrodes. The electrodes were 2 mm in diameter and 12 mm apart and placed on the center of the masseter muscles after palpating it by asking the participant to clench their teeth. The skin at the placement of the electrode was rubbed with alcohol (*Dax Alcogel, Sweden*) and the electrodes were coated with electrode gel (*Cefar Blågel, Sweden*) and attached firmly to the skin with double-sided adhesive tapes and secured with Adhesive medical tape (*Leukoplast, Apotea, Sweden*).

During the experimental session, the participants were asked to eat (chew and swallow) a piece of the test food naturally/habitually as they would eat any other food while their jaw movement in the three dimensions and EMG from the masseter muscles were continuously recorded. The EMG signals were sampled at a rate of 3.2 kHz, while the vertical and lateral positions of the lower jaw relative to the upper jaw were sampled at 800 Hz. All recorded signals were acquired and analyzed using the SC/ZOOM, a custom-made micro-computer-based data acquisition and analysis system (SC/ZOOM, v.3.1.02, Umeå University, Physiology Section, IMB, Umeå, Sweden). During the test the participants were specifically instructed to hold the test food between their tongue and palate, keeping their mouth closed and teeth in the inter-cuspal position (this served as the reference point for kinematic analysis). The examiner signaled the participant to begin chewing. Once they finished chewing and swallowing, participants were asked to return to the inter-cuspal position. Further, the participants were asked to raise their hand and indicate that they had swallowed the test food. The measurements from the jaw movements and EMG recordings (stored offline) were used to identify the number of chewing strokes and the duration of the chewing sequence before swallowing. The onset of the chewing was identified by the changes in vertical dimension and the swallowing was identified by no change in the vertical dimension during chewing. The chewing cycle was defined by an opening phase followed by a closing phase and an occlusal phase which were identified by specific markers as shown in detail in the previous studies (28, 31). Further, the EMG activity of the jaw muscles during the jaw opening, jaw closing, and occlusal phase were also calculated. Thus, the chewing behavior was analyzed by quantifying by number of chewing cycles and duration of chewing sequence when consuming the hard viscoelastic test food and evaluating the different phases of jaw movements and jaw muscle activity during the task.

### Data processing

The signals from the jaw tracker were analyzed using a customized computer-based data analysis program (WinZoom, Umeå University, Physiology Section, IMB, Umeå, Sweden). The comprehensive analysis of chewing behavior involved assessing the distinct phases within the chewing sequence and chewing cycle. In particular, the analysis focused on three segments of each chewing sequence: the beginning, middle, and the end. Each segment was represented by three consecutive cycles. The first and last cycles of the sequence were excluded due to high intra-individual variance across trials. Thus, the second to fourth chewing cycles represented the beginning of the sequence, while the second to fourth cycles from the end represented the end of the chewing sequence.

### Statistical analysis

The assumptions of normality for each parameter were checked using the Shapiro-Wilks test and by visual inspection of QQ plots. The descriptive statistics for the parameters are presented as means and standard deviations. Weight, height, BMI, and performance in food comminution fulfilled the normality criteria and were analyzed using the student *t*-test. The chewing frequency [time per one chewing cycle (Hz)] was calculated by dividing the total duration of chewing by the total number of chewing cycles during a chewing sequence. The number of chewing cycles and duration of chewing and chewing frequency were not normally distributed and compared with the Mann-Whitney *U* test. The jaw movement durations during the beginning, middle, and end sequences were analyzed with two-way ANOVA (Analysis of variance model). The data were first log-transformed and then used in the analysis. The factors in ANOVA were 1. groups (2 levels; anorexia nervosa group and reference group) and 2. phases of the chewing sequence (3 levels; beginning, middle, and end). All conducted tests were two-tailed, with a *P*-value of less than 0.05 considered statistically significant.

## Results

All participants, except for one from the anorexia nervosa group, successfully completed all the tests. The one participant who did not complete the masticatory behavior test refused to eat the test food without citing any reason. There were significant differences in the age (*P* = 0.006) body weight (*P* = 0.002) and BMI (*P* < 0.001) but not height (*P* = 0.515) between the anorexia nervosa group and reference groups. The participants in the anorexia nervosa group were younger and had lower body weight and BMI compared to the reference group (Table 1). The data related to EMG could not be used for the majority of the individuals and therefore was excluded from the analysis.

**Table 1:** Showing mean and standard deviation (SD) of the demographic variables of the participants in the anorexia nervosa group and reference group.

Demographic variables	Anorexia nervosa group (Mean + SD)	Reference group (Mean + SD)	*P*-value
Age	20.2 ± 5.9	23.6 ± 0.9	*P* = 0.006*
Weight	45.5 ± 8.4	61.9 ± 8.0	*P* = 0.002*
Height	165.3 ± 7.8	163.0 ± 3.8	*P* = 0.515
BMI	16.5 ± 3.0	23.3 ± 3.0	*P* < 0.00*

* Significant.

### Chewing performance

**Figure 1:**
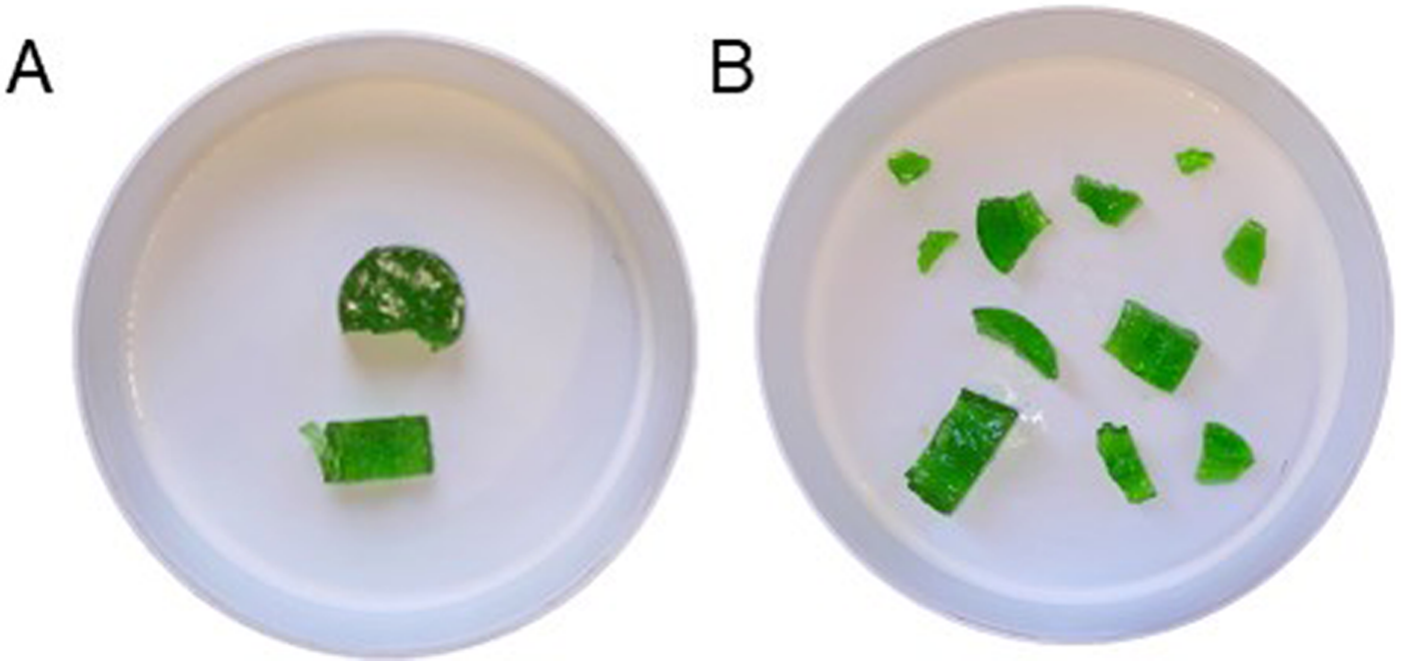
A representative illustration of the expectorated test food from the food comminution test after 10 chewing cycles in **(A)** anorexia nervosa group and **(B)** the reference group of individuals without anorexia nervosa.

**Figure 2:**
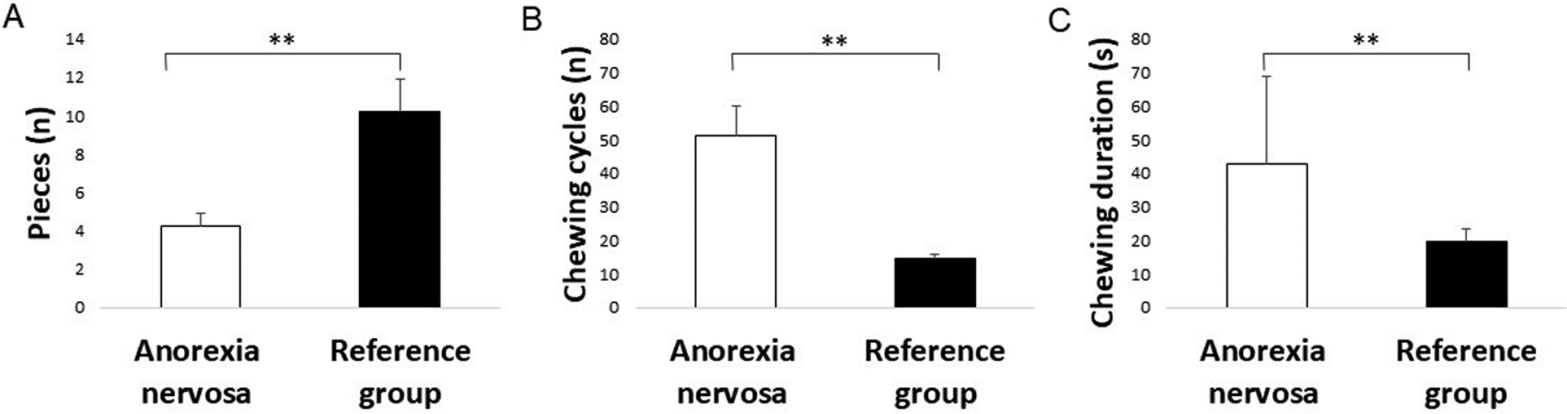
Bar graphs showing the mean and standard errors of mean of the **(A)** number of pieces during the food comminution test, **(B)** number of chewing cycles, and **(C)** duration of chewing sequence (in seconds) in the anorexia nervosa group and reference group. Asterisk (_*_) indicates significant differences.

The results of the chewing performance test showed significant differences in the food comminution test between the two groups. Specifically, the anorexia nervosa group significantly showed fewer pieces in the food comminution test compared to the reference group (*P* = 0.007) (Figures 1, 2). Furthermore, there was a significant difference in the number of chewing cycles (*P* = 0.014), and the duration of the chewing sequence (*P* = 0.004) but no significant difference in the chewing frequency (*P* = 0.054) between the two groups (Figure 2). Overall, the anorexia nervosa group chewed more and for a longer duration yet maintained the same frequency but performed poorly in the food comminution test compared to the reference group.

### Chewing behavior

**Figure 3:**
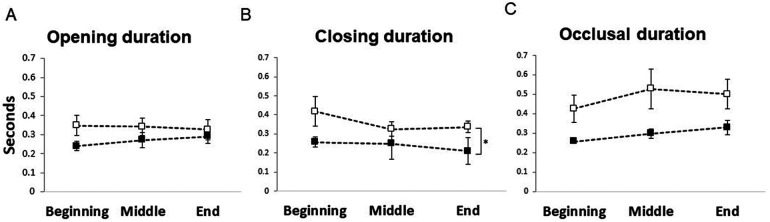
Line graph showing mean and standard error of the mean for **(A)** jaw opening, **(B)** jaw closing and **(C)** occlusal duration (in seconds) from the beginning, middle, and end of the chewing sequence for the anorexia nervosa group (black squares) and reference group (white squares).

The results of ANOVAs on the different parameters of masticatory behaviors showed no significant main effects of either group (*P* = 0.063) or phases (*P* = 0.058) on the occlusal duration of the chewing cycle. Similarly, there was no significant effect of either the groups (*P* = 0.314) or phases (*P* = 0.849) on the jaw opening duration of the chewing cycle. Further, there was also no significant interaction between the groups and phases for either the occlusal (*P* = 0.819) or the jaw opening (*P* = 0.321) duration of the chewing cycle. However, there was a significant main effect of groups (*P* = 0.021) but no significant main effect of phases (*P* = 0.113) and no significant interaction (*P* = 0.549) between groups and phases for the jaw-closing phase of the chewing cycle (Figure 3). The post-hoc analysis of the duration of jaw-closing phase showed significantly longer jaw closing duration in the anorexia nervosa group compared to the reference group (*P* = 0.021).

## Discussion

The current study reports the preliminary findings from a group of patients diagnosed with anorexia nervosa and undergoing care. According to the results of the study, despite having a higher number of chewing cycles and longer chewing durations, anorexia nervosa patients performed significantly worse on the food comminution test compared to the reference group. The results of the analysis of the chewing behavior also showed a significantly longer jaw-closing duration of a single chewing cycle between the anorexia nervosa and reference group. However, there was no significant difference in the jaw opening and occlusal duration between the two groups. The results of the study indicate that people with anorexia nervosa may have difficulty chewing food which is evident in poor chewing performance and also may hold the food longer as reflected in more chewing cycles and longer chewing duration. It is also inferred that these participants tend to spend more time biting through the food exhibited in the longer jaw-closing phase compared to the reference group. As mentioned above, previous studies have reported a significantly higher incidence of dental caries, dental erosion, self-reported dentin hypersensitivity, periodontal disease, xerostomia, and oral mucosa-related complications in patients with eating disorders (15–18). It has also been reported that eating disorder patients report dissatisfaction with their chewing ability, speech, and aesthetic appearance of teeth (32). However, to the best of our knowledge, the functional aspects of chewing, including masticatory performance and masticatory behaviors, have not been examined in previous studies. Therefore, this brief report could offer valuable insights into the oral health and oral functions of individuals with eating disorders.

Studies have used viscoelastic test food to investigate the functional aspects of chewing, providing valuable insights into oral physiology, dietary adaptations, and clinical implications for oral rehabilitation (25, 33). Hence, in the current study, a hard viscoelastic test food with controlled rheological properties was used to test the food comminution ability and masticatory behavior (22–24). Hard viscoelastic test foods are complex food morsels and people with impaired chewing function or impaired oral sensorimotor regulation such as those with dental implants often tend to perform poorly in these tests (22, 23, 28). Although the patient groups in the previous studies (22, 23) and the current study differ, a direct comparison cannot be made. However, the current study's results indicate that the anorexia nervosa group performed poorly in the food comminution test compared to the reference group, despite chewing the test food almost twice as long and having nearly twice the number of chewing cycles. Additionally the longer jaw-closing duration of a single chewing cycle between the anorexia nervosa and reference group implies that these people perhaps had difficulty in biting through the test food.

Anorexia nervosa is characterized by extreme restriction of calorie intake, an intense fear of weight gain, and a distorted body image (34). Studies have reported atypical eating behaviors in anorexia nervosa patients (35, 36). As mentioned above, participants in anorexia nervosa group chewed the test food longer before swallowing and consumed the test food with more chewing cycles. The reason could be that people with anorexia nervosa have a preoccupation with food, constant thoughts about food, and a strong desire to control their eating (37, 38). It has been particularly reported that these patients have a slow eating pace and often tend to chew and spit food (39, 40). Therefore, our preliminary findings in the present study (partially) support these observations. It can be speculated that patients perhaps frequently prolong their eating duration by chewing on food and retaining food morsels in their mouths.

In the current study, the detailed analysis of the mastication process was based on the understanding that chewing, like other motor tasks, relies on well-coordinated movements (41). Each segment/phase in the chewing sequence requires precise timing and coordination, especially when manipulating food morsels in the oral cavity (27, 42). To gain a comprehensive understanding of these dynamics, we divided mastication into beginning, middle, and late segments, with further subdivisions into each chewing cycle's opening, closing, and occlusion phases. This approach enables us to capture subtle variations in chewing function, emphasizing the significance of segment and phase-specific analysis in understanding chewing dynamics (25). Comparing cycle times and phase durations between groups can provide valuable insights, as variations often indicate underlying neuromuscular or structural issues. For instance, our previous research has shown that the timing and coordination of jaw movements are vital for understanding the neurophysiology of chewing behavior in people with dental implants (25), altered oral sensory inputs (27, 28), or children in different developmental stages (24). By analyzing each phase in detail, we aim to enhance diagnostic and therapeutic strategies tailored to diverse populations, improving the efficiency and effectiveness of mastication.

Clinical studies frequently encounter limitations that should be acknowledged to ensure the accuracy of interpretations and to guide future research. One such limitation is the relatively small sample size and lack of sample size calculation for investigating the preliminary hypothesis. It was also evident that participants in the study group were younger than the reference group. However, previous studies have suggested that children as young as 15–16 years can perform most oral tasks like adults and they develop a more “adult-like” behavior (43). It is therefore unlikely that an age difference of about 3–5 years can affect the performance of oral motor tasks. Further, a notable limitation of the current pilot study was the technical issues encountered with the EMG recordings. Specifically, the EMG activity from a majority of the participants could not be analyzed due to noise, which may have resulted from interference or artifacts that obscured the true signal. Furthermore, for a significant portion of the remaining participants, the data were incomplete, reducing the sample size and limiting the reliability of any conclusions that could be drawn from the EMG results. As a result, we excluded this data from our analysis. Future studies should consider these technical challenges to ensure the quality and reliability of EMG data. Overall, the current study is a pilot attempt, and the results may be preliminary indications for developing a stronger hypothesis for future longitudinal studies. Therefore, the results of the current study should be treated with caution.

This current study within its limitations raises important questions about the relationship between decreased masticatory performance, changes in chewing behavior, and anorexia nervosa. Due to the small number of participants, it was not possible to perform a robust statistical analysis to directly correlate these variables with the severity of the disorder. It was also observed that the anorexia nervosa group had a longer jaw-closing duration for each chewing cycle compared to the reference group. As mentioned above this suggests that individuals with anorexia nervosa experience more difficulty biting through the hard test food. It is plausible that the reduced masticatory performance and longer jaw-closing duration observed in the study could be linked to the extent of muscle atrophy and overall physiological deterioration often seen in cases of anorexia nervosa (44, 45). This could potentially reduce masticatory bite force or occlusal forces leading to reduced masticatory performance. Although this is not specifically studied in the current study future studies may include bite force as a measurement to describe the physiological state of the masticatory system. The clinical implications of this pilot study indicate that patients with anorexia nervosa may not only have a preoccupation with food but also show signs of poor masticatory performance and altered chewing behavior. This could affect their ability to process food effectively. Recently we have shown that the type of dentition and masticatory performance could affect the dietary habits and nutritional status in older individuals (46). Therefore, the findings from the current study highlight the importance of considering masticatory function and associated masticatory behavior in the management and rehabilitation of anorexia nervosa patients. Future studies should investigate the relationship between decreased masticatory performance, changes in chewing behavior, and the severity of anorexia nervosa. Additionally, future research should explore the underlying mechanisms by which factors such as muscle atrophy, nutritional deficiencies, and altered eating habits contribute to the development and progression of anorexia nervosa. Understanding the nuances of oral function in clinical evaluations can help healthcare providers tailor interventions more effectively. This insight is particularly valuable when planning support strategies, such as dietary recommendations and feeding management, especially for patients with anorexia nervosa.

## Conclusion

Anorexia nervosa patients showed poor performance in the food comminution test, increased chewing cycles, and longer chewing duration compared to the reference group of young healthy participants. In addition, the results also showed longer jaw-closing duration in the patient group but no significant differences in jaw opening, and occlusal duration between the two groups. Further research is needed to better understand the underlying factors contributing to impaired masticatory performance in patients with eating disorders and to develop effective interventions to address these issues.

## Data Availability

The raw data supporting the conclusions of this article will be made available by the authors, without undue reservation.
